# Opto‐Electrical Properties of Group IV Alloys: The Inherent Challenges of Processing Hydrogenated Germanium

**DOI:** 10.1002/advs.202200814

**Published:** 2022-05-06

**Authors:** Thierry de Vrijer, Bilal Bouazzata, Ashwath Ravichandran, Julian E. C. van Dingen, Paul J. Roelandschap, Koos Roodenburg, Steven J. Roerink, Federica Saitta, Thijs Blackstone, Arno H. M. Smets

**Affiliations:** ^1^ Photovoltaic Materials and Devices TU Delft Mekelweg 4 Delft 2628CD The Netherlands

**Keywords:** chemical stability, germanium tin GeSn, group IV alloys, hydrogenated germanium Ge:H, plasma enhanced chemical vapor deposition

## Abstract

In this paper the opto‐electrical nature of hydrogenated group IV alloys with optical bandgap energies ranging from 1.0 eV up to 2.3 eV are studied. The fundamental physical principles that determine the relation between the bandgap and the structural characteristics such as material density, elemental composition, void fraction and crystalline phase fraction are revealed. Next, the fundamental physical principles that determine the relation between the bandgap and electrical properties such as the dark conductivity, activation energy, and photoresponse are discussed. The unique wide range of IV valence alloys helps to understand the nature of amorphous (a‐) and nanocrystalline (nc‐) hydrogenated (:H) germanium films with respect to the intrinsicity, chemical stability, and photoresponse. These insights resulted in the discovery of i) a processing window that results in chemically stable Ge:H films with the lowest reported dark conductivity values down to 4.6·10^‐4^ (Ω ·cm)^‐1^ for chemical vapor deposited Ge:H films, and ii) O, C and Sn alloying approaches to improve the photoresponse and chemical stability of the a/nc‐Ge:H alloys.

## Graphical abstract

The opto‐electrical nature of 400+ PECVD processed hydrogenated group IV films is studied. The fundamental relations between the bandgap, conductive behavior, and structural material characteristics are revealed. The unique wide range of IV‐valence alloys helps to understand the nature of a/nc‐Ge:H films with respect to the intrinsicity, chemical stability, and photoresponse, resulting in the identification of a suitable processing window.

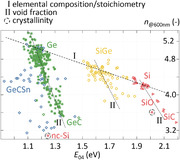

## 1 Introduction

Semiconductors based on group IV elements are the workhorse of the modern electronics industry. Elements like silicon, carbon, and germanium are used for a range of applications in the fields of micro‐electronics,^[^ ^1^ ^]^ optics,^[^ ^2^ ^]^ photonics,^[^ ^3^, ^4^ ^]^ and photovoltaics (PV).

There are some inherent advantages to the processing of group IV alloys. Group IV elements can form tetrahedral structures, with each valence electron covalently bonded, requiring no other element for neutral, intrinsic processing. This is unlike other major semiconductor technologies which require elements from different groups for intrinsic processing. Examples are semiconductors based on elements from groups III and V and chalcogenides, which use elements from groups II‐VI or I‐III‐VI. These elements have different properties; different radii, electronegativity, and melting points, which inherently introduces complexity. This complexity is predominantly expressed in two ways.

First, processing chemically stable materials is fundamentally more challenging. The relation between the complexity of the global chemical system and chemical stability of chalcogenides, for example, has been well documented.^[^ ^5^, ^6^, ^7^, ^8^ ^]^ A similar struggle for chemical stability is ongoing in the relatively novel field of the chemically complex perovskites.^[^ ^9^, ^10^, ^11^, ^12^ ^]^ A result of the inherent chemical instability of these materials are either potential trade‐offs between performance and stability, or more complex and expensive encapsulation on a device level.

Secondly, the inherent complexity is expressed in the processing of these materials. Processing is generally accomplished by exerting great control, using techniques such as molecular beam epitaxy or metal‐organic chemical vapor deposition, used for the processing of III‐V's, or atomic layer deposition which is used for the processing of certain layers in perovskite solar cells.^[^ ^13^, ^14^ ^]^ Alternatively, if deposition techniques are used during which less control is exerted, such as the evaporation‐based techniques or sputtering used for chalcogenides, a high temperature anneal step is often required for obtaining a device grade material.^[^ ^6^, ^7^, ^15^, ^16^ ^]^ Either route can result in time and/or energy intensive, and therefore relatively expensive, processing.

Additionally, with the expected upscaling of certain semiconductor applications, such as PV, in mind, there is elemental abundance to consider. Si and C are among the 12 most abundant elements in the lithosphere, in terms of molar abundance, with Si ranked second.^[^ ^17^ ^]^ Ge and Sn are also more abundant than elements used for alternative PV technologies, such as indium and arsenic used for III‐V's, cadmium, tellurium, and selenide used for chalcogenides and elements such as cesium, rubidium, iodine, and bromine used for metal halide perovskites.^[^ ^10^, ^18^, ^19^ ^]^

In light of the inherent advantages, a range of plasma enhanced chemical vapor deposition (PECVD) processed hydrogenated (:H) group IV alloys are presented in this work, demonstrating a wide range of bandgap energies. These alloys are based on carbon, silicon, germanium and tin and their alloys with oxygen. The fundamental relation between material elemental composition, density, and the opto‐electrical properties of the group IV alloys are discussed, based on the 400+ films presented in this work. In particular, the processing of a low bandgap group IV Ge:H material is discussed, in reference to other group IV alloys. Based on our experience processing over 230 Ge:H films, three main challenges are identified, related to the material intrinsicity, stability, and photoresponse. Finally, two potential pathways are discussed for tackling these challenges.

## 2 Experimental Section

The Ge:H and GeCSn:H films are simultaneously processed on 4 inch, 500µm thick monocrystalline silicon wafers cut in quarters for Fourier Transform Infrared (FTIR) spectroscopy measurements and on 10cm x 2.5cm Corning Eagle XG glass for all other measurements. The Ge:H and GeCSn:H films were processed in the Cascade RF‐PECVD reactor, which has a circular electrode with a diameter of 160 mm and an electrode spacing that is varied between 10 and 20mm. Cascade is a laminar flow reactor, where germane (GeH_4_) and molecular hydrogen are used as precursor gasses. In addition to these precursors, tetramethyltin (TMT) (Sn(CH_3_)_4_) is used for the GeCSn:H depositions. The TMT, a liquid at room temperature, is evaporated in a separate canister at 70 °C. For a fraction of the GeCSn:H samples the TMT flow was diluted in He. Injection of TMT into the reactor is controlled through a valve, similar to those used for atomic layer deposition. For the samples presented in this work, the open time of the valve was varied between 5 and 10 ms while the close time was varied between 100 ms and 60 s. The ratio of the close time to the open time is referred to as the duty cycle. More detailed experimental information regarding the GeCSn:H films is reported elsewhere.^[^ ^20^ ^]^

All other films are processed in a separate PECVD reactor, with a flat 12 cm x 12 cm shower‐head electrode, on 10 cm x 2.5 cm Corning Eagle XG glass. The films presented in this work have a thickness of 80–180nm. The deposition conditions of the processed films, including the applied radiofrequenci power (*P*_RF_), depostion pressure (*p*), substrate temperature (*T*_S_) and precursor gas flow rates in standard cubic centimeters per minute (sccm), are reported in **Table** **1**.

**Table 1:** Deposition conditions of the films presented in this work

	**P_RF_**	**p**	**T_S_**	**SiH_4_**	**GeH_4_**	**CH_4_**	**Sn(CH_3_)_4_**	**H_2_**	**CO_2_**
	[mW·cm^−2^)	[mbar]	[°C]	(sccm)	(sccm)	(sccm)	(duty cycle (‐))	(sccm)	(sccm)
SiC:H	20.8	3.6	180	5		10		200	
SiO:H	20.8	3.6	180	5				200	5
a‐Si:H‐1^a)^	69.3	9	130	1.5–5				200	
a‐Si:H‐2^a)^	18–20.8	0.7	130–180	40					
nc‐Si:H^b)^	277.8	4	180	3.3				120	
SiGe:H	13.9–56	2–6	150–210	30	0.4–5.3			150‐200	
GeO:H	20.8	3.6	180		2–4			200	20‐70
GeC:H	20.8	3.6	180		2	20–60		200	
Ge:H	12.4–248.7	0.5–6	200–350		0.5–2			100‐200	
GeCSn:H	14.9–29.8	1–5	210–290		1–2		20–12000	200	
best a‐Ge:H	14.9	4	290		2			200	
best nc‐Ge:H	24.8	1	290		1			200	

a) No distinction is made between the two sets of conditions in the following figures;

b) Processed at 40 MHz.

FTIR spectra were obtained using a Thermo Fisher Nicolet 5700 spectrometer. The FTIR spectra were fitted using the Fityk freeware.^[^ ^21^ ^]^ The background was subtracted manually. The absorption coefficient of the summed Ge‐O_x_ and Ge‐C_x_ bonds in the 650–1000cm^‐1^ wavenumber range (α_tot_) is used in this work as a thickness independent metric for sum of the relative Ge‐O and Ge‐C peak intensities. The method for obtaining this metric, as well as examples of representative FTIR and Raman measurements, are reported elsewhere.^[^ ^22^ ^]^

**Figure 1:**
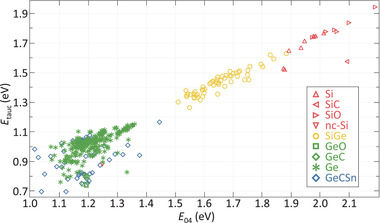
Cody–Lorentz bandgap energy as a function of the *E*_04_ optical bandgap energy of a range of hydrogenated group IV alloys

The thickness, real part of the refractive index at a wavelength of 600nm (*n*_@600nm_) and optical bandgap energies were determined through spectroscopic ellipsometry (SE). The SE measurements were fitted using a Cody–Lorentz model. Determining the electrical bandgap energy (*E*_G_) of the amorphous and nano‐crystalline stoichiometric and non‐stoichiometric hydrogenated group IV alloys presented in this work is a non‐trivial and potentially arbitrary process. For that reason, the optical bandgap energy (*E*_04_) is used in this work to describe trends in the optical absorption of the films. *E*_04_ was determined by calculating the photon energy at which the absorption coefficient equals 10^4^cm^‐1^. Based on mean square error analysis, the *E*_04_ has an error margin of ±0.004 eV. Additionally, the Cody–Lorentz optical bandgap energy (*E*_C‐L_ ) was obtained from the fitted SE model. The relation between *E*_04_ and *E*_C‐L_ is shown in **Figure** **1**, from which it can be observed that the trend, as a function of elemental composition, is largely similar and that the *E*_C‐L_ is roughly 0.2 eV lower than *E*_04_. The larger spread for the Ge:H films and alloys is likely a results of the large degree of heterogeneity between samples in terms of oxidation and carbisation states and amorphous and crystalline (c‐) material phase fractions.

For the dark‐ and photo‐conductivity measurements, parallel 500 nm Al electrodes were evaporated onto the films. The dark conductivity at room temperature (*σ*_d_) was determined by measuring the current at a fixed voltage of 10 V, in a dark environment, at different temperatures ranging from 130°C to 60°C in 5°C decrements. From this measurement the activation energy (*E*_act_) of the films was also calculated, using the Arrhenius equation. The photoconductivity (σ_ph_) was calculated by measuring the current at a fixed voltage of 10V, using an AM1.5G solar simulator at an illumination of 100 mW·cm^−2^ and a controlled temperature of 25 °C. For the σ_d_ and σ_ph_ measurements, 4 different sets of contacts were measured on a single film. The resulting dark conductivity values showed a maximum error margin of about ±11%, while the *E*_act_ values showed a maximum error margin of ±9 meV.

## 3 Why Different Group IV Alloys

**Figure 2:**
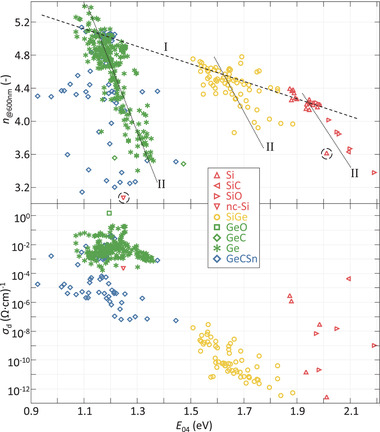
*n*_@600nm_ (top) and σ_d_ (bottom) as a function of the *E*_04_ optical bandgap energy of a range of hydrogenated group IV alloys. Trendlines **I** and **II** in top plot are referenced in text. GeO samples represented by black squares in top plot for improved identification.

With the hydrogenated group IV alloys presented in this work a wide range of *E*_04_ are achieved, ranging from about 0.9 eV for the high *n*_@600nm_ Ge:H films to about 2.2 eV for the low *n*_@600nm_ SiO:H film, as presented in **Figure** **2**. The refractive index can be considered a metric for material density, as was previously demonstrated for hydrogenated silicon.^[^ ^23^ ^]^ Consequently, the films with the highest density are those with the lowest bandgap energy.

The opto‐electrical properties of hydrogenated group IV elements are the result of the structural characteristics of the films, which in turn are a result of the parameters like the substrate type used for deposition, deposition technique and deposition condition. In this section, the high level relations between the structural and opto‐electrical characteristics are addressed. For the films presented in Figure 2 more information about the relation between the processing conditions and structural characteristics, as well as more detailed structural investigation of the films, is provided for GeCSn,^[^ ^20^ ^]^ Ge,^[^ ^22^, ^24^, ^25^, ^26^ ^]^ SiGe,^[^ ^22^, ^27^ ^]^ SiO.^[^ ^24^ ^]^

From these collective works, three structural characteristics through which a change in density can be realized. For two of these mechanisms trendlines have been added in Figure 2. The first effect, indicated by trendline **I**, involves the elemental composition. The use of larger, higher‐period elements will result in lower bandgap energies. This effect, long established in III‐V semiconductors,^[^ ^28^, ^29^ ^]^ occurs because larger elements generally results in larger averaged bond lengths (lattice constants) resulting in smaller overlap of the orbital of the valence electrons. As a consequence, splitting of the sp3 anti‐bonding and bonding level, the chemical origin of the bandgap, is reduced and bandgap energies are decreased. Considering group IV elements, it can be observed that the bandgap energy of SiC > Si > SiGe > Ge > GeCSn. Similar, more detailed, observations have been reported before in relation of the stoichiometry of two‐element group IV alloys such as SiGe:H^[^ ^27^, ^30^, ^31^ ^]^ and SiC:H.^[^ ^32^, ^33^ ^]^

The second set, trendlines **II**, indicate a change in the *n*_@600nm_ and *E*_04_ as a function of void fraction, or porosity. In these hydrogenated amorphous (a‐) materials, nano‐sized voids are present in the material at sites where multiple C, Si or Ge atoms are missing. We have reported before on the relation between void fraction and bandgap energy in Ge:H and SiGe:H^[^ ^22^ ^]^ as well as in a‐Si:H.^[^ ^34^ ^]^ It should be noted that the relation between the void fraction, or porosity, and *E*_04_ in amorphous materials is often reported as a relationship between the hydrogen concentration and the bandgap energy. Indeed, the two properties are often related; as the void fraction is increased more dangling bond sites are available for hydrogen to attach to. Moreover, the void fraction is often determined using specific hydrogen vibrational modes as a metric.^[^ ^22^, ^23^, ^30^, ^35^, ^36^ ^]^ However, using a‐Si:H it was demonstrated^[^ ^37^ ^]^ that for similar hydrogen concentration dissimilar void fractions, bandgap energies and absorptive behavior can be realized. The volumetric compressive nature of the amorphous matrix as a function of the density, distribution and size of nano‐sized voids determines the absorptive nature, and by extension the bandgap energy, of amorphous materials.^[^ ^34^ ^]^ The fraction of dangling bonds occupied by hydrogen, and consequently the hydrogen concentration, will influence this compressive nature. The notion that trendlines **II** in Figure 2 is the result of a change in hydrogen concentration would therefore be an oversimplification.

Finally, the third mechanism for realizing a change in *E*_04_ involves the crystalline phase fraction. This is essentially an extension of the previous observation, as the crystalline phase is the most dense bonding configuration for atoms and consequently has the lowest bandgap energy. This can be observed considering the dashed circles in Figure 2 (top), where the nanocrystalline (nc‐) Si sample at around 1.25 eV has a bandgap energy of about 0.75 eV below that of its porous a‐Si:H counterpart. The presence of a crystalline phase results in a heterogeneous material in which the amorphous phase and crystalline phase each retain their respective opto‐electrical properties, making interpretation more challenging.

In Figure 2 (bottom), the σ_d_ can be observed to decrease with increasing *E*_04_. In fact, the σ_d_ of the most silicon rich SiGe:H samples and Si:H samples is about 10 orders of magnitude lower than that of most of the Ge:H samples. This relation is not unexpected. The σ_d_ is a result of the charge carrier concentration and mobility. At a given temperature, the intrinsic carrier density (*n*_i_) will increase with decreasing *E*_G_, as described in Equation (1). Low *E*_G_ materials will therefore have a higher σ_d_. Alternatively, the relation between the *E*_G_ and σ_d_ can be understood through Equation (2). For intrinsic materials, where *E*_act_ ≈ 0.5*E*_G_, the σ_d_ will exhibit an exponential increase with decreasing *E*_G_. Despite this inherent relation between *E*_04_ and σ_d_, for the GeCSn:H films σ_d_ values are obtained that are 2‐3 orders of magnitude below that of the Ge:H films, for similar *E*_04_. Considering the Ge:H sample size and extent of variations in processing conditions, this indicates some inherent challenges in the processing of Ge:H films.

(1) $$n_{i}^{2}∼\exp\left(\frac{-E_{G}}{k_{b}T}\right)$$

(2) $$\sigma_{d}=\sigma_{0}\exp\left(\frac{-E_{\mathrm{act}}}{k_{b}T}\right)$$

## 4 The Challenges of Processing Ge:H

### 4.1 Intrinsicity of Ge:H

**Figure 3:**
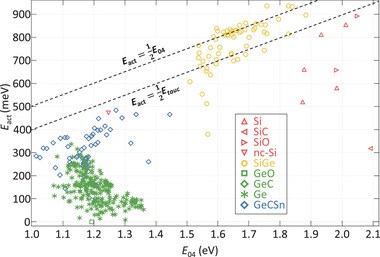
*E*_act_ as a function of the *E*_04_ of a range of hydrogenated group IV alloys. Trendlines are added to indicate an intrinsic material, based on the *E*_04_ and *E*_C‐L_. For the *E*_C‐L_ trendline the assumption *E*_C‐L_=*E*_04_‐0.2eV is used, based on data presented in Figure 1.

Ideally, group IV elements used as absorber materials in p‐i‐n or n‐i‐p junctions should be intrinsic. In intrinsic materials the concentration of electrons and holes are roughly equal. If the concentration of one charge carrier type exceeds the other, the recombination probability of minority charge carriers increases and consequently the probability of successful electron‐hole pair collection decreases. It should be noted that for optimal operation, when used in a PV device, the absorber could be made slightly p‐type to compensate for the lower hole mobility in reference to the electron mobility for these amorphous materials.^[^ ^38^ ^]^ The activation energy of the group IV alloys are shown in **Figure** **3**. The *E*_act_ in this work is defined as the difference between the Fermi level and the nearest band edge. An intrinsic material is one where the Fermi level is positioned halfway the bandgap, so with *E*_act_ ≈ 0.5*E*_G_. Two lines are plotted in Figure 3, indicating the *E*_act_ of an intrinsic film based on the *E*_04_ and *E*_C‐L_. It can be observed that not all of the Si(Ge,O):H films are intrinsic. The non‐intrinsic behavior in these undoped films is the result of the energetic nature of the defects and has been linked to the fraction of nano‐sized voids in the material and the hydrogen concentration.^[^ ^39^, ^40^ ^]^ Additionally, the elemental composition of the alloys can influence the type and presence of majority charge carriers.^[^ ^36^, ^41^ ^]^ Nonetheless, for all Si(Ge,O):H alloys, films are presented that either are intrinsic, or very close to intrinsic. This is unlike Ge:H. None of the 200+ Ge:H films, processed under a wide range of conditions, are intrinsic. Rather, the films have an n‐type nature.^[^ ^24^, ^42^, ^43^ ^]^

### 4.2 Photoresponse of Ge:H

**Figure 4:**
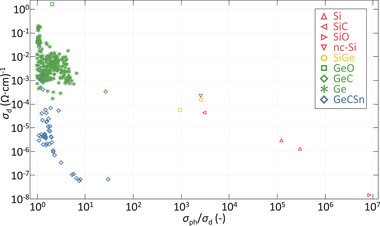
σ_d_ as a function of the σ_ph_/σ_d_ ratio, of a range of hydrogenated group IV alloys

An additional challenge is related to the low σ_ph_/σ_d_ of the Ge:H films in reference to the other group IV alloys. The σ_ph_/σ_d_ ratio of the Ge:H films does not exceed a value of 5, as can be observed in **Figure** **4**. This is in line with the few other reports of CVD processed Ge:H films the authors are aware of, where the σ_ph_/σ_d_ ratio did not exceed 1.1.^[^ ^31^, ^44^ ^]^

The low σ_ph_/σ_d_ is largely a fundamental effect. As discussed earlier, upon a decrease of *E*_G_ the σ_d_ is increased exponentially. If we assume intrinsic materials with *E*_act_ = 0.5*E*_G_ and *E*_G a‐Si_ = 1.8, and *E*_G a‐Ge_ = 0.9eV, the $\exp(\frac{-E_{\mathrm{act}}}{k_{b}T})$ term in Equation (2) would increase by 8 orders of magnitude. σ_ph_, which is basically σ_d_ augmented by conductivity that is the result of photo‐generated charge carriers, will undergo a similar increase. Additionally, when *E*_G_ is decreased, a larger fraction of photons can be absorbed from the solar spectrum, which affects the photoconductivity. This effect is less and less however, when the *E*_G_ is further decreased, since the solar spectral irradiance decreases exponentially for *E*_G_ ⩾1.82 eV, as described by Planck's law. If, for example, the *E*_G_ is decreased from that of a‐Si:H to that of a‐Ge:H, the photon flux available in the AM1.5g solar spectrum is increased from 1.3×10^21^s^−1^·m^−2^ to 3.3×10^21^s^−1^·m^−2^, by a factor of about 2.5. As the exponential increase of both σ_ph_ and σ_d_ far exceeds the individual near‐linear increase of σ_ph_ by orders of magnitude, the ratio of the σ_ph_ to σ_d_ will decrease strongly with decreasing *E*_G_. The low σ_ph_/σ_d_ of Ge:H can fundamentally be understood to be lower than that of Si(Ge,C,O):H. It is unlikely however that σ_ph_/σ_d_ is fundamentally limited to the degree observed in Figure 4 and.^[^ ^31^, ^44^ ^]^ Improvement could potentially be achieved through improved intrinsicity and decreased defect density, as will be discussed in Section 5.1.

### 4.3 Stability of Ge:H

A final challenge is related to the chemical stability of the Ge:H films. It was demonstrated in earlier work that for the majority of the processed samples, for a very large part of the processing window, a signature in the FTIR spectra appeared within minutes after deposition, that is related to the post‐deposition oxidation of the Ge:H films.^[^ ^24^ ^]^ We argued that this post‐deposition reaction is related to the microscopic properties of the Ge:H films. In subsequent work, we found that post‐deposition not only an oxidation reaction occurred, but also a carbisation reaction,^[^ ^22^, ^26^ ^]^ which likely involves some form of catalytic CO_2_ reduction. We demonstrated that the rate of oxidation is directly related to the density of the material and specifically the fraction of nano‐sized voids in the material.^[^ ^25^ ^]^ These collective works have also provided some insight into possible routes to processing stable Ge:H films with favorable opto‐electrical properties.

## 5 Potential Solutions for Processing Ge:H

In processing over 230 Ge:H films, using the wide range of deposition conditions reported in Table 1, three main challenges were identified. These challenges for processing device quality Ge:H are related to the intrinsicity, photoresponse, and stability of the films. In this section, two potential pathways are considered to resolve these challenges. The first pathway concerns a very specific processing window, while the second involves alloying of Ge:H.

### 5.1 The Right Processing Window

**Figure 5:**
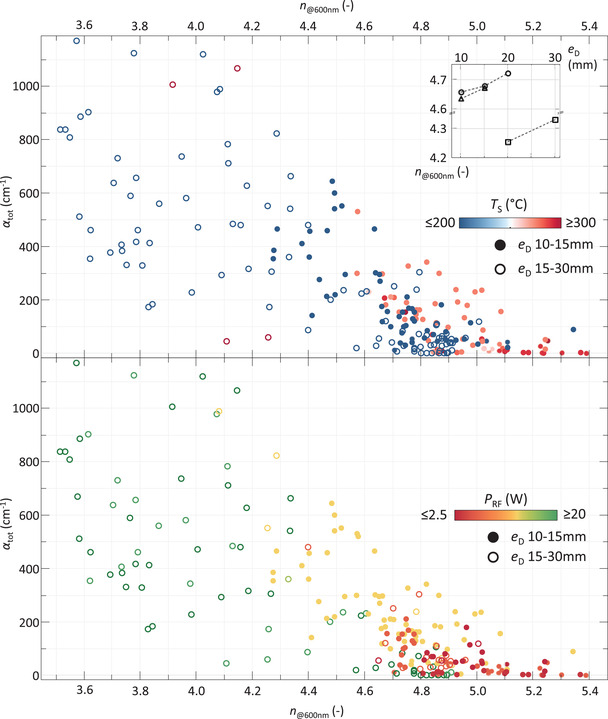
α_tot_ as a function of *n*_@600nm_ for all Ge:H films. Closed icons and open icons indicate samples processed with *e*_D_ ⩽15mm and *e*_D_>15mm, respectively. Color indicates *T*_S_ (top) and *P*_RF_ (bottom). Inset shows *n*_@600nm_ as a function of *e*_D_ for three sets of samples, processed under different conditions. Deposition conditions within each set, indicated by the dashed lines, are kept constant.

In **Figure** **5** the *n*_@600nm_ is plotted as a function of α_tot_, the metric used to indicate chemical instability in the processed films. As expressed in Section 2, α_tot_ is a film‐thickness independent metric, obtained from infrared measurements, that indicates the absorption by oxide and carbide bonds that are formed post‐deposition. The figure shows that with increasing *n*_@600nm_, with increasing material density, the stability of the material is increased, as reported earlier.^[^ ^22^, ^24^, ^25^ ^]^ More importantly, the figures show that a decrease of the electrode distance (*e*_D_), an increase of *T*_S_ and a decrease of the *P*_RF_ each result in the processing of denser, and consequently more stable, films. Moreover, the most stable films, with α_tot_ ≈0 and *n*_@600nm_ ⩾5.2 are processed when all three conditions are met.

The relation between increased deposition *T*_S_ and improved stability is one we reported on in more detail in earlier work.^[^ ^22^ ^]^ In that work, a mechanism was proposed in which temperature induced densification is achieved through a process that involves the retroactive restructuring and densification of the (sub‐)surface region during material growth, under influence of a high temperature plasma. The region undergoing restructuring has been referred to as the growth zone.^[^ ^45^, ^46^ ^]^ Growth zone densification is likely the result of the virtual movement of small voids toward the surface upon continued plasma exposure, and has earlier been observed amorphous silicon.^[^ ^47^, ^48^ ^]^

A clear correlation can be observed between the *e*_D_ and the chemical stability. Two observations can be made in this regard. The first observation is related to the fraction of samples with *n*_@600nm_ ⩽4.4, which are predominantly samples processed at *e*_D_ ⩾15 mm. This observation is likely related to the fact that a much larger deposition parameter space (e.g., a wider range of *P*_RF_, *p* and precursor gas flows) was explored at *e*_D_ ⩾15 mm, resulting in a larger fraction of samples processed at sub‐optimal conditions and more porous unstable films.

The second observation is related to the highest *n*_@600nm_ values obtained for stable films, with α_tot_ roughly ⩽50cm^‐1^, at *e*_D_ ⩾15 mm with respect to those obtained at *e*_D_<15 mm. The largest *n*_@600nm_ at larger *e*_D_ is just over 4.9, while that of a smaller *e*_D_ is almost 5.4. This relation between α_tot_ and *n*_@600nm_ will be addressed in more detail further along this section.

The relation between the *e*_D_ and increased *n*_@600nm_ is an indirect one. For three sets of samples where *e*_D_ was decreased with all other conditions kept constant, instead of an increase, *n*_@600nm_ was observed to decrease slightly with decreasing *e*_D_, as can be observed in the inset in Figure 5 (Top). Smaller *e*_D_ allows for processing at lower *P*_RF_ and higher pressure, in a regime referred to as the high pressure depletion regime.^[^ ^49^ ^]^ It is apparent from Figure 5 that processing at relatively low power is a prerequisite for dense and stable Ge:H films.

As to the relation between *P*_RF_ and *n*_@600nm_, there are three main ways in which *P*_RF_ can affect material growth. The first two are related to precursor gas dissociation. An increase of deposition power results in more effective precursor gas dissociation and consequently in a larger GeH_x_ growth flux. This generally results in a higher deposition rate and more porous growth. Additionally, if more than one precursor gas is used, the balance of radicals in the growth flux could be upset by a change in deposition power. Stronger bonds require more energy for dissociation. As a consequence, with increasing *P*_RF_ the dissociation of stronger bonds is increased, in relative terms, in reference to the dissociation of weaker bonds. For Ge:H processing H_2_ and GeH_4_ are used. The former has a bond dissociation energy of 436 kJ/mol,^[^ ^50^ ^]^ while breaking the bond of a single hydrogen atom from the latter only takes about 340–360 kJ/mol.^[^ ^51^, ^52^, ^53^ ^]^ A relative increase of atomic hydrogen in the growth flux, in reference to GeH_x_ radicals, can therefore be expected with increasing *P*_RF_.

Both these mechanisms can also be realized through changes in other deposition conditions. An overall growth flux increase can be realized through an increase of *p* and changes in the growth flux composition can realized by varying the ratio of the germane flow rate (*F*_GeH4_) to the hydrogen flow rate *(F*_H2_). Both of these mechanisms undoubtedly play a role. Indeed in earlier work an improvement of the stability of Ge:H films was demonstrated by tuning the ratio of the precursor gasses *F*_GeH4_/*F*_H2_, and *p*.^[^ ^24^ ^]^ However, the most dense and stable films in Figure 5, with *n*_@600nm_ ⩾5, are processed at a relatively large range of *p*, from 1 to 4 mbar, and *F*_GeH4_/*F*_H2_, from 0.25% to 1%. As these two conditions are not indicative of a dense film, and *P*_RF_ is, the relevant mechanism in this range is likely energetic ion‐bombardment. The processing of dense films under low‐energetic ion‐bombardment conditions is not in line with earlier reports on Ge:H^[^ ^44^ ^]^ and SiGe:H^[^ ^27^ ^]^ films, where the beneficial effect ion‐bombardment induced densification was reported.

From this we conclude that the most dense Ge:H films are processed under conditions that result in low‐energetic ion bombardment, where densification is achieved through temperature induced restructuring of the growth zone. These conditions are facilitated by a small electrode distance, as a power‐threshold is required for a stable plasma and a smaller electrode facilitates a stable plasma at lower powers.

**Figure 6:**
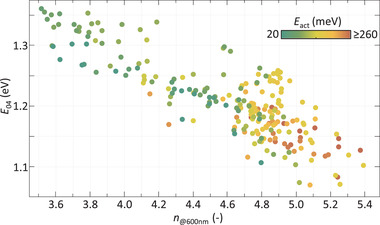
*E*_04_ as a function of *n*_@600nm_ for all Ge:H films. Color indicates *E*_act_.

A relation has been established between the *n*_@600nm_, and by extension the material density, and stability of Ge:H films. In addition to being stable, the films should be intrinsic and have a low *E*_G_. In **Figure** **6** the relation between *n*_@600nm_, *E*_04_ and *E*_act_ is presented for all Ge:H films. A decrease of *E*_04_ can be observed with increasing *n*_@600nm_ for the Ge:H films, similar to the trend observed in Figure 2 for all hydrogenated group IV alloys. The *E*_act_ can be observed to generally increase with increasing *n*_@600nm_. This trend is somewhat counter‐intuitive, since the difference between an intrinsic Fermi energy level and the nearest band‐edge logically decreases with decreasing *E*_G_. The increasing *E*_act_ with increasing *n*_@600nm_ is the result of two reactions. First, the activation energy of Ge:H films decreases upon post‐deposition oxidation,^[^ ^24^ ^]^ as the GeO_x_ has a strong n‐type nature.^[^ ^54^ ^]^ This effect is predominantly visible for samples with *n*_@600nm_ ⩽4.9. Additionally, in earlier work it was demonstrated that the n‐type defect density, and consequently *E*_act_, scales with the concentration of nano‐sized voids in Ge:H.^[^ ^22^ ^]^ A smaller concentration of nano‐sized voids therefore results in denser films with higher *E*_act_, despite lower *E*_04_.

Only for samples with *n*_@600nm_ ⩾5.2, in the absence of the post‐deposition oxidation and negligible void fractions, is the intuitively understood decrease of *E*_act_ with *E*_04_ reflected. The decrease of *E*_04_ in this range is predominantly the result of variations in the crystalline phase fractions, as discussed in more detail elsewhere.^[^ ^22^ ^]^

**Figure 7:**
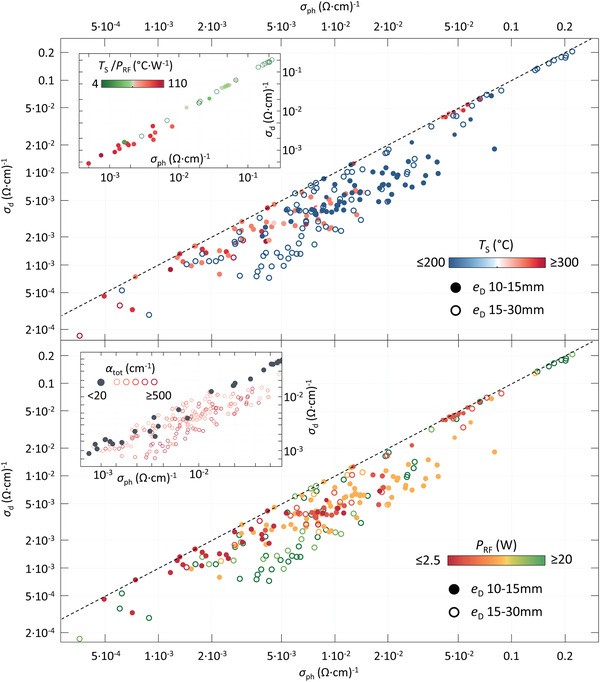
σ_d_ as a function of σ_ph_ for all Ge:H films. Closed icons and open icons indicate samples processed with *e*_D_ ⩽15 mm and *e*_D_>15 mm, respectively. Color indicates *T*_S_ (top) and *P*_RF_ (bottom). The top inset exclusively shows stable films (α_tot_<20), while colors indicate *T*_S_/*P*_RF_. In bottom inset the colors and icon type indicate α_tot_.

The right processing conditions, reported in Table 1, yield dense and stable a/nc‐Ge:H films. Additionally, it can be observed that the σ_d_ generally decreases with decreasing *P*_RF_ and increasing *T*_S_. This effect can predominantly be observed in the inset of **Figure** **7** (top), in which unstable films are omitted. The dashed lines in Figure 7 indicate the position where σ_ph_/σ_d_=1. The conductivity values of most films are positioned near this line, indicating that σ_ph_ scales with σ_d_. Surprisingly, the improved σ_d_ for the dense Ge:H films σ_d_ does not result in higher σ_ph_/σ_d_.

Low σ_d_ values are realized by stable as well as oxidized Ge:H films. Moreover, the largest σ_ph_/σ_d_ ratios, those that deviate most from the dashed line, are achieved by oxidized Ge:H samples, as can be observed in the inset of Figure 7 (bottom). The higher photoresponses, and relatively low σ_d_ for certain oxidized samples, are achieved despite much lower *E*_act_.

The σ_d_ can be considered a function of the defect density, represented by the recombination parameter σ_0_ in Equation (2), and the position of the defect energy levels with respect to the nearest band edge, represented by *E*_act_. The fact that low σ_d_ values are achieved for oxidized Ge:H samples, with much lower *E*_act_ than the stable Ge:H samples, must mean that even the densest Ge:H samples have a relatively high defect density. A defect density that is much decreased in the oxidized Ge:H samples. This effect, a reaction in which oxygen introduced in the material post‐deposition results in the passivation of defects, decreasing the defect density as well the *E*_act_, has been discussed in more detail in earlier work.^[^ ^24^ ^]^

A relatively large defect density in the Ge:H films would also explain the poor photoresponse of the Ge:H films and improvement of σ_ph_/σ_d_ upon oxidation. The low photoresponse indicates that only a small fraction of photo‐generated charge carriers are collected. This means that the carriers recombine between the generation and collection event. The recombination process is a defect‐assisted recombination process, considering the amorphous nature of the materials. A decrease of the defect density would consequently facilitate an increase of the σ_ph_ with respect to the σ_d_.

In amorphous silicon, hydrogen is responsible for the passivation of defects. The germanium films in this work are hydrogenated and the Ge:H films with *n*_@600nm_ ⩾5.0 have a hydrogen concentration in the range of 3–5%. This is in the same range as the hydrogen concentration in relatively dense Si:H films.^[^ ^23^, ^55^ ^]^ If the concentration of hydrogen in both materials is similar, why then is the degree of defect passivation not? A potential explanation is provided by studies concerning the nature of defects in c‐Ge and the behavior of hydrogen in c‐Ge. Several studies show that the dangling bonds in Ge, unlike in silicon, are exclusively negatively charged.^[^ ^56^, ^57^ ^]^ Interstitial hydrogen in germanium is also exclusively negatively charged,^[^ ^56^, ^57^ ^]^ or at least never positively charged,^[^ ^58^ ^]^ which is also unlike silicon. As a consequence, hydrogen passivation of the germanium dangling bond is very ineffective. It should be noted that these studies are performed on c‐Ge, not on a/nc‐Ge:H. Nevertheless the described behavior is in good agreement with the trends observed in this work, related to the hydrogen concentration and defect passivation behavior of hydrogen in Ge:H inferred from the σ_d_ and σ_d_ trends. Additionally, the results suggest that oxygen, unlike hydrogen, readily passivates the Ge dangling bonds.

Considering i) the poor passivation behavior of atomic hydrogen in Ge:H, ii) the observed change in conductive behavior upon oxidation of Ge:H films and iii) the increase of *E*_act_ for denser Ge:H films, a decrease in defect density is the most likely cause for changes in σ_d_ in chemically stable a‐Ge:H films. A low σ_d_ can therefore be considered indicative of good intrinsic a‐Ge:H films. It should be noted that the lowest σ_d_ values reported in Figure 7 are 1‐5 orders of magnitude lower than those reported for all other known a/nc‐Ge:H films,^[^ ^41^, ^42^, ^59^, ^60^, ^61^, ^62^ ^]^ processed by chemical vapor deposition techniques, to the best of the authors knowledge.

### 5.2 GeX Alloys

The notion that oxygen is able to passivate defects in Ge:H allows for another approach to tackling the intrinsicity, stability and photoresponse related challenges in the processing of Ge:H, namely alloying. The results of the preceding section indicate that through alloying, through the introduction of elements or compounds that can passivate Ge‐defects, the photoresponse can be improved. Similar improvements to the photoresponse can be observed through alloying with carbon and silicon, although these effects can at least partly be ascribed to the relation between *E*_G_, *E*_act_, and σ_ph_/σ_d_, described in Section 4.2, as alloying with O,C and Si inevitably results in an increase of *E*_G_.

**Figure 8:**
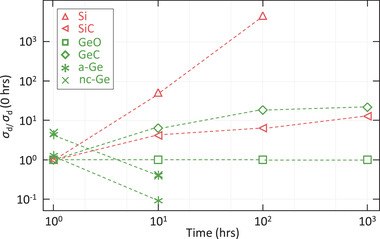
Development of the σ_d_ for a range of group IV alloys as a function of time stored in de‐ionized water. Atomic C fraction in GeC and SiC ≈7 to 9%. Atomic O fraction in GeO ≈3 to 4%

To investigate the influence of Ge:H alloying on the stability, a number of GeX:H and SiX:H alloys were stored in de‐ionized (DI) water and the evolution of the opto‐electrical properties were monitored over time. The electrical properties, which proved most sensitive to degradation, of the films are presented in **Figure** **8**. The figure shows σ_d_ of the films, measured after 1‐10‐100‐1000 h in DI water, in reference to the initial measurement prior to water exposure. It can be observed that a decrease of the σ_d_ upon water exposure occurs exclusively for the a/nc‐Ge:H samples. For Si:H and the different alloys σ_d_ increases over time. This indicates that exclusively for a/nc‐Ge:H the oxidation improves the electrical characteristics of the films, as discussed in.^[^ ^22^, ^24^ ^]^ Moreover, the a/nc‐Ge:H films are fully etched in DI water in the 10–100 hrs period. The reactions involved in the Ge:H consumption and the relation between material density, etch rate and type of reaction are discussed in detail elsewhere.^[^ ^25^ ^]^

Figure 8 also shows that the stability of the Ge:H films is strongly improved when alloyed with O and C. A similar improvement of the stability of σ_d_ can be observed in the Si:H films, when alloyed with C. The Ge(C,O):H films are no longer consumed within the 1000 hrs measured time‐frame and σ_d_. In fact, the σ_d_ of GeO:H remains very stable over time, while GeC:H continuously increases, with about an order of magnitude over the measured time‐frame. While Ge(C,O):H alloys exhibit higher *E*_04_ than unalloyed Ge:H, a similar improvement in chemical stability could potentially be achieved in GeSn(C):H alloys, for which lower bandgap energies can potentially be realized. Consequently, potential pathways toward the successful processing of GeSn(C):H are currently being investigated.^[^ ^20^ ^]^

## 6 Conclusion

In this work over 400 PECVD processed films are presented, based on hydrogenated group IV alloys, exhibiting an *E*_04_ range of over 1.3 eV. Based on these results the fundamental relations between the material density, as a function of the elemental composition, void fraction and crystalline phase fraction, and bandgap energy are discussed. Moreover, the relation between the bandgap energy and electrical materials characteristics such as the dark conductivity, activation energy, and photoresponse are considered.

Additionally, based on 230 a/nc‐Ge:H films, 3 inherent challenges are identified related to the PECVD processing of hydrogenated germanium. These are related to the intrinsicity, chemical stability and photoresponse of the films. Two pathways for tackling these challenges are presented. The first involves a very specific processing window, at relatively high temperature and low RF power at a small electrode distance. Using this processing window chemically stable Ge:H films are processed with σ_d_ values down to 4.6×10^‐4^ (Ω ·cm)^−1^ and σ_ph_/σ_d_ values up to 2.3, which are the best achieved for chemical vapor deposition processed Ge:H films, to the best of the author's knowledge. The second pathway involves the alloying of Ge:H. Improved chemical stability is demonstrated for Ge:H films alloyed with O and C. While these alloys exhibit higher *E*_04_ than unalloyed Ge:H, a similar improvement in chemical stability could be achieved in GeCSn:H alloys, for which *E*_C‐L_ values as low as 0.7 eV are demonstrated.

## Conflict of Interest

The authors declare no conflict of interest.

## Data Availability

The data that support the findings of this study are available from the corresponding author upon reasonable request.
